# Integrating multi-scale data for a network model of macaque visual cortex

**DOI:** 10.1186/1471-2202-14-S1-P111

**Published:** 2013-07-08

**Authors:** Maximilian Schmidt, Sacha van Albada, Rembrandt Bakker, Markus Diesmann

**Affiliations:** 1 Institute of Neuroscience and Medicine (INM-6) and Institute for Advanced Simulation (IAS-6), Jülich Research Centre and JARA, Jülich, Germany; 2 Donders Institute for Brain, Cognition and Behavior, Radboud University Nijmegen, the Netherlands; 3 Faculty of Medicine, RWTH Aachen University, Aachen, Germany; 4 RIKEN Brain Science Institute, Wako-Shi, Japan

## 

Models of cortical dynamics usually either cover small cortical circuits in detail, or represent large patches in a highly simplified manner, for instance using a few differential equations for each area. This is due partly to limited computational resources, and partly to sparsity of large-scale structural connectivity data. These two barriers are now being lifted. Recent developments in the simulation technology NEST [1] and the availability of supercomputers like JUQUEEN in Jülich and the K computer in Japan make it possible to represent substantial parts of primate cortical tissue at cellular and synaptic resolution. At the same time, gaps in experimental knowledge on large-scale connectivity are gradually being closed [2].

Making use of these developments, we construct a model comprising the 32 areas of the macaque cortex associated with visual processing, where the individual areas are based on our recent layered cortical microcircuit model [3]. While this model accounts for the distribution of spike rates across layers and populations, it is heavily underconstrained because only about half the synapses onto the cells in the microcircuit have a local origin. The extension to multiple areas makes it possible to account for a large majority of the synapses and thereby enhances the self-consistency of the model. We compile a realistic structural connectivity map by incorporating multiple datasets from both electro-physiological [4] and anatomical studies [2,5] on different species. The map is adapted to the macaque using realistic area- and layer-specific neuron densities and in-degrees. Both local and inter-area connectivity are layer-specific. The CoCoMac database [5] provides the basis for the inter-area connectivity, and is supplemented with quantitative information from further tracing studies [2]. We complete these data sets by applying empirical connectivity rules, for instance exploiting the dependence of connection strengths on inter-area distances, and of laminar connection patterns on architectural type differences [6].

Combining a simple neuron model with complex connectivity enables us to study the influence of the structural connectivity itself on cortical dynamics. We present preliminary results on basic dynamical features like area- and population-specific firing rates and degrees of irregularity. In addition, we examine the influence of the cortico-cortical connections by comparing dynamical features like power spectra of the isolated primary visual cortex (V1) with Poisson input to its dynamics when embedded in the global network. This model bridges the gap between abstract large-scale models and small-scale microcircuit models. It therefore provides a link between global activity patterns and local spiking activity.
